# Unproductive Effects of ALK Gene Amplification and Copy Number Gain in Non-Small-Cell Lung Cancer. ALK Gene Amplification and Copy Gain in NSCLC

**DOI:** 10.3390/ijms21144927

**Published:** 2020-07-12

**Authors:** Federica Zito Marino, Gerardo Botti, Gabriella Aquino, Stefano Ferrero, Gabriella Gaudioso, Alessandro Palleschi, Danilo Rocco, Rosario Salvi, Maria Carolina Micheli, Pietro Micheli, Alessandro Morabito, Gaetano Rocco, Antonio Giordano, Rossella De Cecio, Renato Franco

**Affiliations:** 1 Department of Mental and Physical Health and Preventive Medicine, Pathology Unit, University of Campania “L. Vanvitelli”, 80138 Naples, Italy; federica.zitomarino@unicampania.it; 2 Pathology Unit, Istituto Nazionale per lo Studio e la Cura dei Tumori, IRCCS “Fondazione Pascale”, 80131 Naples, Italy; g.botti@istitutotumori.na.it (G.B.); g.aquino@istitutotumori.na.it (G.A.); r.dececio@istitutotumori.na.it (R.D.C.); 3 Division of Pathology, Fondazione IRCCS Ca’ Granda-Ospedale Maggiore Policlinico, 20122 Milan, Italy; Stefano.ferrero@unimi.it (S.F.); gabriella.gaudioso@unimi.it (G.G.); 4 Department of Biomedical, Surgical and Dental Sciences, University of Milan, 20100 Milan, Italy; 5 Thoracic Surgery and Lung Transplant Unit, Fondazione IRCCS Ca’ Granda Ospedale Maggiore Policlinico, 20122 Milan, Italy; alessandro.palleschi@policlinico.mi.it; 6 Department of Pulmonary Oncology, AORN Dei Colli Monaldi, 80131 Naples, Italy; danilorocc@yahoo.it; 7 Thoracic Surgery Unit, AORN Dei Colli Monaldi, 80131 Naples, Italy; rosdoc@libero.it; 8 Pathology Unit, AORN Dei Colli Monaldi, 80131 Naples, Italy; mariacarolinamicheli@gmail.com (M.C.M.); pietromicheli1@virgilio.it (P.M.); 9 Thoracic Medical Oncology, Istituto Nazionale per lo Studio e la Cura dei Tumori, IRCCS “Fondazione Pascale”, 80131 Naples, Italy; a.morabito@istitutotumori.na.it; 10 Department of Thoracic Surgery, Memorial Sloan Kettering Cancer Center, New York, NY 10065, USA; roccog@mskcc.org; 11 Department of Medicine, Surgery and Neuroscience, University of Siena, 53100 Siena, Italy; antonio.giordano@unisi.it; 12 Sbarro Health Research Organization, Philadelphia, PA 19122, USA

**Keywords:** ALK, amplification, copy number gain, non-small-cell lung cancer

## Abstract

**Background:** The Anaplastic Lymphoma Kinase (ALK) gene is known to be affected by several genetic alterations, such as rearrangement, amplification and point mutation. The main goal of this study was to comprehensively analyze *ALK* amplification (*ALK*-A) and *ALK* gene copy number gain (*ALK-CNG*) in a large cohort of non-small-cell lung cancer (NSCLC) patients in order to evaluate the effects on mRNA and protein expression. **Methods:** *ALK* locus number status was evaluated in 578 NSCLC cases by fluorescence in situ hybridization (FISH). In addition, ALK immunohistochemistry and ALK mRNA in situ hybridization were performed. **Results:** Out of 578 cases, 17 cases showed *ALK*-A. In addition, 14 cases presented *AL*K-CNG and 72 cases presented chromosome 2 polyploidy. None of those carrying *ALK*-A and -CNG showed either ALK immunohistochemical expression or ALK mRNA expression through in situ hybridization. We observed a high frequency of extra copies of the *ALK* gene. **Conclusions**: Our findings demonstrated that *ALK*-A is not involved in mRNA production and consequently is not involved in protein production; these findings support the hypothesis that *ALK*-A might not play a role in the pathogenesis of NSCLC, underlining the absence of a specific clinical application.

## 1. Introduction

Anaplastic lymphoma kinase (ALK) is a transmembrane tyrosine kinase receptor. During embryogenesis, ALK is expressed in the nervous system, but its expression decreases after birth [1]. In anaplastic large cell lymphoma (ALCL), ALK was originally characterized as a nucleophosmin fusion partner [2].

*ALK* rearrangement (*ALK*-R) has been also reported in 3–7% of all non-small-cell lung cancers (NSCLCs). *ALK* fusion proteins activate many different overlapping pathways, such as the Ras/Raf/MEK/ERK1/2 pathway, the Janus-activated kinase (JAK)/signal transducer and activator of transcription (STAT) pathway, the phosphatidylinositol 3-kinase (PI3K)/Akt (PKB) pathway and the phospholipase C (PLC)-γ pathway [3,4].

NSCLC patients harboring *ALK*-R present a unique subgroup of clinical and pathological features that are responsive to treatment with specific inhibitors [5].

The *ALK* gene could be a fusion partner of several genes in chromosomal rearrangements, but it could also be involved in other genetic alterations such as mutations and amplification; in specific clinical settings, it is responsible for gene deregulation and thus constitutive activation of the receptor [6].

Among these alterations, *ALK* gene amplification (*ALK*-A) has been identified in various cancers such as ALCL, rhabdomyosarcoma, carcinoma of the esophagus, adult renal cell carcinoma and hepatocellular carcinoma [7].

*ALK*-A is a frequent additional genetic mechanism in the tumorigenesis of neuroblastoma [8]. In addition, evidence from in vitro studies has shown that cell lines with *ALK*-A are sensitive to specific inhibitors, suggesting a potential therapeutic application in *ALK*-A patients [9].

*ALK*-A was also found to be a common aberration in inflammatory breast cancer (IBC) cell lines and tissue samples [10]. In vivo studies have reported sensitivity of IBC xenograft models with *ALK*-A to crizotinib; on the basis of these preclinical data, clinical trials for *ALK*-A IBC patients are currently in progress [11].

Previous results have shown that *ALK*-A is also a common occurrence in esophageal cancer, with comparable rates in both squamous cell carcinoma and adenocarcinoma. However, ALK immunohistochemical expression was not observed in such series [12].

In NSCLC, the data reported for *ALK*-A are scant, although ALK deregulation through gene rearrangement represents the basis for targeted therapy. In a large series of NSCLC patients, a high frequency of *ALK*-A has been reported, showing a significant correlation with epidermal growth factor receptor (EGFR) gene amplification [13].

Furthermore, *ALK*-A has also been described in lung sarcomatoid carcinomas as an early, not random, clonal event closely associated with polysomy of chromosomes 7 and 17 [14]. On the basis of this biological background, *ALK*-A could represent a clinical frontier to extend the spectrum of NSCLC patients sensitive to specific ALK inhibitors.

The main aim of this study was to comprehensively analyze *ALK*-A and *ALK* gene copy number gain (*ALK*-CNG) in a large cohort of NSCLC patients to identify a possible association with RNA expression and protein overexpression.

## 2. Results

### 2.1. Clinical and Pathological Characteristics of Patients

Overall, 578 NSCLC patients were analyzed. Among these, 398 had adenocarcinomas (ADCs), 139 had squamous cell carcinomas (SQs), 5 had adenosquamous lung carcinomas (AdSqLCs) and 36 had large-cell carcinomas. The clinical and pathological features of these patients are summarized in Table 1.

As many as 330 of the 578 patients were current smokers, while 83 had never smoked and 32 were ex-smokers; no information on smoking habits was available for 133 patients (23.0%). Postoperative pathological stages of lung carcinomas were IA in 132 cases (22.8%), IB in 120 (20.8%), IIA in 188 (32.6%), IIB in 23 (4.0%), III in 2 (0.3%) and IV in 81 (14.0%); the pathological stage was not available for 32 cases (5.5%).

Of the 578 NSCLCs, 37 were grade I (6.4%), 262 were grade II (45.3%), 210 were grade III (36.3%) and 2 were grade IV (0.3%); grades were not available for 67 cases (11.6%).

### 2.2. Fluorescence In Situ Hybridization

**Figure 1:**
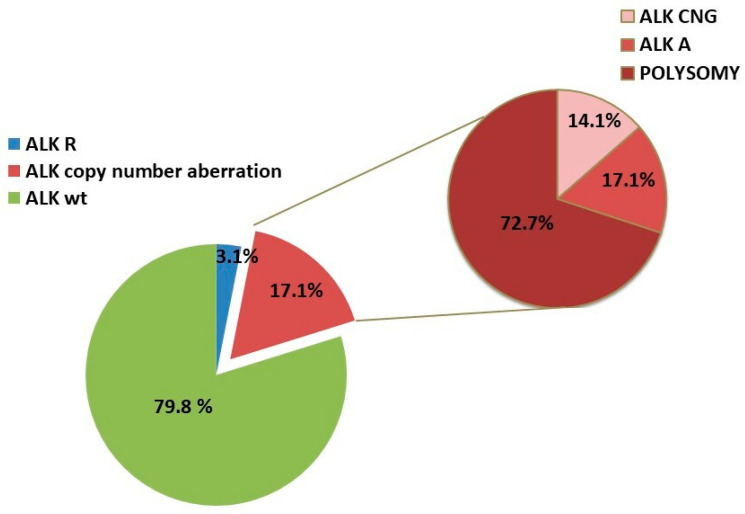
Anaplastic Lymphoma Kinase (ALK) and centromeric probe 2 (CEP2) fluorescence in situ hybridization (FISH) results in our series.

**Figure 2:**
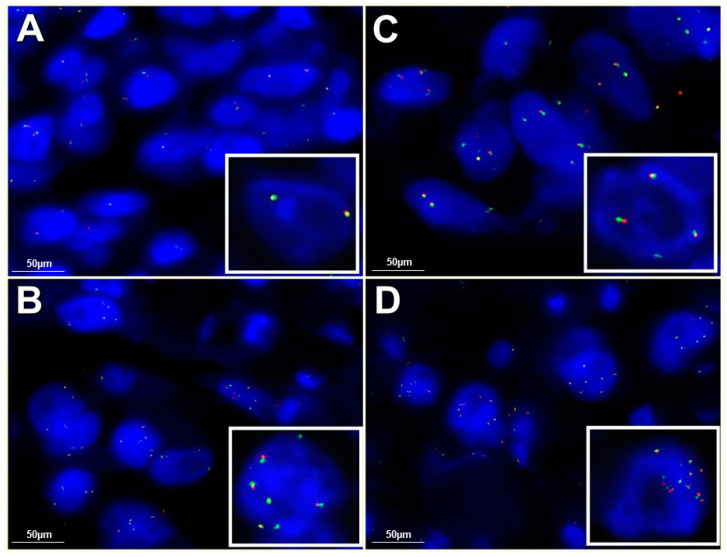
FISH *ALK* results: (**A**) FISH *ALK* wild-type (100× magnification); (**B**) FISH *ALK* gene copy number gain (*ALK*-CNG) (100× magnification); (**C**) FISH *ALK* amplification (*ALK*-A) with ≥6 signals/cell (100× magnification); (**D**) FISH *ALK*-A with 8–12 signals/cell (100× magnification).

**Figure 3:**
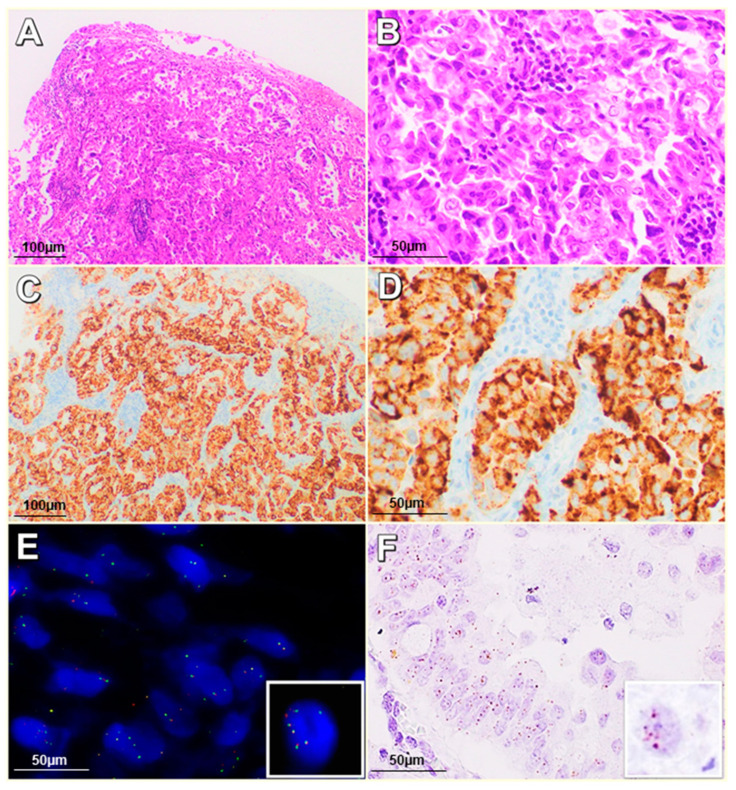
Case harboring *ALK* rearrangement (*ALK*-R) and *ALK*-A: (**A**,**B**) Hematoxylin and Eosin (H&E) staining (20–40× magnification); (**C**,**D**) ALK immunohistochemistry (IHC) positive (20–40× magnification); (**E**) ALK FISH with *ALK*-R and *ALK*-A (63× magnification); (**F**) ALK ISH mRNA expression, average 4–6 dots/cell (60× magnification).

*ALK*-R was harbored by 18 patients (3.1%) (Figure 1). *ALK*-A was shown in 17 of the 578 patients; in particular, 12 patients showed 6 signals and 5 patients showed a number of signals ranging from 8 to 12 per nucleus (Figure 2). Only 1 patient showed concomitant *ALK*-R and *ALK*-A (Figure 3).

The clinical and pathological features of all patients carrying *ALK*-A are detailed in Table 2. No statistical correlation between ALK amplification and patients’ clinical and pathological features was found.

Of the 578 cases (17.1%) 99 showed an increased number of *ALK* gene copies; however, after corrections with a centromeric probe 2 (CEP2) control probe, 72 cases (12.5%) with mean copy number for the *ALK* locus (*ALK* LSI)/CEP2 ratio of <2 were considered to have chromosome 2 polyploidy. Therefore, there were 14 real *ALK*-CNG cases (2.4%). No statistically significant association was found between *ALK*-CNG and clinical or pathological parameters. As many as 506 out of 578 cases presented a disomy of chromosome 2.

### 2.3. Immunohistochemistry Analysis of ALK Protein Expression

Specimens from *ALK*-R patients were immunostained by the anti-ALK D5F3 clone. None of the patients carrying *ALK-*CNG and *ALK*-A displayed ALK expression, regardless of the anti-ALK clone utilized (D5FE3, ALK1, 5A4). Only the case carrying concomitant *ALK*-R and *ALK*-A showed ALK expression, likely due to the rearrangement rather than the amplification (Table 3).

### 2.4. In Situ Hybridization (ISH) Assay for ALK RNA Detection

**Figure 4:**
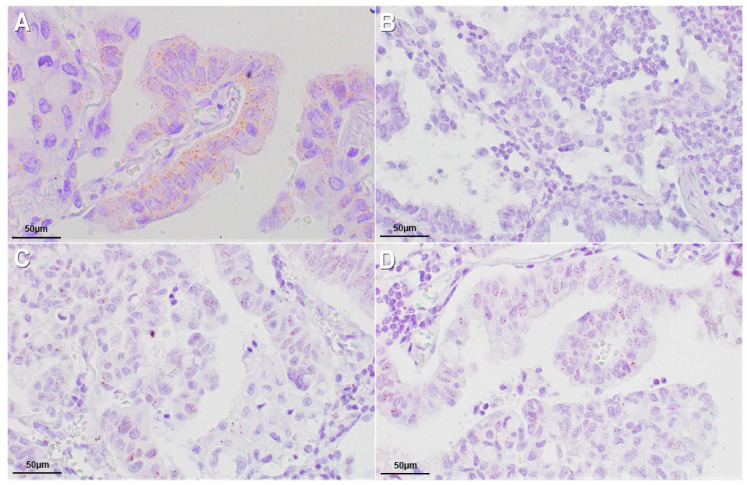
ALK ISH results: (**A**) ALK mRNA expression >6 dots/cell in positive control (60× magnification); (**B**) ALK mRNA negative (60× magnification); (**C**) ALK mRNA expression, >2 dots/cell in 10% of neoplastic cells (60× magnification); (**D**) ALK mRNA expression, average 4–6 dots/cell (60× magnification).

All *ALK-*R cases showed ALK RNA expression analyzed through in Situ Hybridization assay. None of the cases carrying *ALK*-CNG and *ALK*-A showed ALK RNA expression (Figure 4). Only the case carrying concomitant *ALK*-R and *ALK*-A showed ALK RNA expression, likely due to the rearrangement rather than the amplification (Table 3).

## 3. Discussion 

*ALK*-R in NSCLC is a target for directed therapy with specific tyrosine kinase inhibitors. Other *ALK* gene alterations, including mutations and amplification, have been identified in various cancer types, although they do not currently represent druggable aberrations in clinical practice. In vitro studies have shown that in NSCLC cell lines carrying *ALK*-CNG are sensitive to ALK inhibitors, although, to the best of our knowledge, a possible role of *ALK*-A has not yet been fully clarified in NSCLC patients. *ALK*-A and -CNG have been studied in different tumor types, not always related to ALK protein overexpression or increased downstream signaling [7,15].

In our study, the potential association between amplification and CNG was analyzed at two levels, particularly through the evaluation of both protein product and mRNA expression.

In our comprehensive evaluation, *ALK*-A was not associated with specific clinical and pathological features or histotype, unlike *ALK*-R, which is generally related to adenocarcinoma, never-smoker status and young age. Moreover, in our cohort, *ALK*-A occurred in NSCLC patients regardless of *ALK*-R, with only one case having concomitant *ALK*-R and *ALK*-A.

The copy number gain of the *ALK* gene in NSCLC can be due to either *ALK*-A or aneusomy for chromosome 2. Therefore, we stratified NSCLC patients in whom *ALK*-CNG was a result of chromosome 2 aneusomy against those with a true ALK-gene copy gain.

As many as 3% of the patients in our series harbored *ALK*-A. Overall, we observed two molecular patterns of *ALK*-A that distinguished cases; i.e., one group had a gene copy number that was increased by an average of 5–6 signals per nucleus (2% of cases), while another group showed an average of 8–12 signals per nucleus (1% of cases). Previous studies have reported the presence of *ALK*-A in NSCLC. Camidge et al. screened 1426 NSCLC clinical specimens (174 *ALK* rearranged and 1252 *ALK* not rearranged) and found an increased native ALK copy number (≥3 copies/cell in ≥40% cells) in 19% of *ALK* rearranged cells and 62% of *ALK* non-rearranged cells [16]. Another study with 170 patients reported a frequency as high as 10% [13]; a more recent study evaluating *ALK* gene status by fluorescence in situ hybridization (FISH) reported that only 2 out of 205 cases harbored *ALK*-A [17].

The variability of the *ALK*-A frequency in previous literature could be ascribable to both the different score systems used for assessing ALK -CNG and the concomitant evaluation of chromosome 2 polyploidy. Salido et al. considered gain to occur when a mean copy number from 3 to 5 fusion signals was observed in more than 10% of cells, and they considered amplification to occur when more than 6 copies of ALK were observed in ≥10% of the cells analyzed [13]. Camidge et al. used a unique cut-off of ≥3 copies per cell in ≥40% of cells [16]. Caliò et al. considered gene amplification as the presence of ≥8 copies of *ALK* per cell [17]. In our series, 72 out of 578 cases firstly showed *ALK*-CNG, although after corrections by the CEP2 control probe, only 14 cases confirmed a real *ALK*-CNG, excluding the chromosome 2 polyploidy cases. In agreement with previous results [16,17], we found that *ALK*-CNG occurred mostly because of chromosome 2 polyploidy (12.5% of cases) rather than as a result of locus-specific gene copy number gains (2.4% of cases). Previously published data reported that the increased copy numbers of the *ALK* gene could also be associated with chromosome 2 aneusomy in IBC, with a frequency of 64% [18].

These findings suggest that chromosome 2 polyploidy is a frequent event in NSCLC, making FISH CEP2 an indispensable tool to discriminate true extra *ALK* gene copies.

Chromosomal instability is a well-known phenomenon in NSCLC biology and is associated with tumor progression [19]. Previous molecular cytogenetic analysis of NSCLC tumors and cell lines by spectral karyotyping and comparative genomic hybridization (CGH) reported that chromosome 2p is frequently involved in gains [19,20,21]. Chromosome 2p gains have been identified through CGH assays in both squamous cell carcinoma (27.5%) and adenocarcinoma (20%) [21]. Moreover, amplifications at chromosome 2p23-p24—the locus where the *ALK* gene maps—have been described in NSCLC tumors and cell lines [20]. Our findings support previous data reported by Caliò et al. [17] confirming that the increase of the *ALK-*CNG is more frequently due to the polyploidy of chromosome 2.

For ALK IHC expression, all cases harboring *ALK*-A were negative regardless of the clones used. Our results are in accordance with previous findings observed in other series of NSCLCs and esophageal cancer reporting negative ALK IHC in *ALK*-A cases [12,13,14]. Despite the high specificity and sensibility of the D5F3 clone in detecting *ALK*-R cases, compared to other common ALK1 and 5A4 clones, the results obtained through IHC and FISH were discordant in some cases, with an unpredictable response to ALK inhibitors [22].

It is worth noting the different sensibility and specificity observed for these clones in detecting *ALK*-R in NSCLC and other tumors, which were higher for D5F3. According to this observation, the use of the clone D5F3 is recommended in clinical practice to select patients harboring *ALK*-R for targeted therapy [23]. The differences observed with the use of the different clones could be related to different rearrangements occurring in NSCLC which are responsible for the loss of specific antigenicity detected by the clones. Thus, the negativity described in our *ALK*-A and *ALK*-CNG cases could be related to the loss of all antigenicities detected by the available clones. There are four ALK antibody clones that have been evaluated for NSCLC. In our study, we also examined ALK mRNA expression in cases of NSCLCs carrying *ALK*-A in order to evaluate the status of gene transcription in *ALK*-A and *ALK*-CNG cases. An intriguing result was that all cases carrying *ALK*-A were negative not only to immunostaining for anti-ALK clones but also to mRNA expression in the ISH assay. The ability to visualize individual transcript molecules within a single cell represents a powerful tool for clarifying the possible value of *ALK*-A in NSCLC. Finally, the absence of ALK mRNA supports the negative ALK IHC results observed in *ALK*-A cases.

## 4. Materials and Methods

### 4.1. Patients and Specimens

A series of 578 NSCLC tumor tissue samples from surgical resections performed between 2006 and 2011 at the Istituto Nazionale per lo Studio e la Cura dei Tumori, IRCCS “Fondazione Pascale”, the Fondazione IRCCS “Ca’ Granda”—Ospedale Maggiore Policlinico and the AORN Vincenzo Monaldi were collected. Sections of 4 µm were obtained from at least 3 blocks per tumor for each case and stained with hematoxylin and eosin. All 578 cases were reviewed according to the new IASLC/ATS/ERS classification [24].

In this study, we analyzed various clinical and pathological parameters, including age of the patient at initial diagnosis, smoking habits, tumor stage and histologic grade and the eventual recurrence or metastasis.

After careful explanation, informed consent was obtained from all patients, which authorized the reexamination of biological samples for research purposes, as approved by the General Directors of the involved hospitals (Istituto Nazionale per lo Studio e la Cura dei Tumori, IRCCS “Fondazione Pascale” and Monaldi Hospital, number 15, approval date 15 January 2016, establishing and regulating our Biobank, the Fondazione IRCCS “Ca’ Granda”—Ospedale Maggiore Policlinico Institutional Review Board 179/2013; approval date 19 March 2013). 

### 4.2. Tissue Microarray Construction

All 578 NSCLCs were used for building 14 tissue microarrays (TMAs) after careful re-evaluation of tumoral and nontumoral areas by two experienced pathologists. The tissue microarrays were built using three cores of 1 mm from different areas and, whenever possible, one core of normal tissue of the same block for each case, collected in a recipient paraffin block (3 × 2.5 cm) using a semi-automated tissue arrayer (Galileo TMA; Integrated Systems Engineering, Milano, Italy) [25].

### 4.3. Fluorescence In Situ Hybridization

FISH analysis was performed on unstained 3-μm-thick TMA slides to detect *ALK* status and CEP2. Two types of probes used: (i) an *ALK* probe-Vysis LSI ALK Dual Color, Break Apart Rearrangement Probe (Abbott Molecular, Abbott Park, Illinois, USA) that is constituted by two different fluorescent probes that flank the *ALK* breakpoint, where one probe is labeled with a fluorochrome in Spectrum orange and the other is labeled with a fluorochrome in Spectrum green, and (ii) a centromeric alpha-satellite specific for chromosome 2-Vysis CEP 2 (D2Z1) ((Abbott Molecular, Abbott Park, IL, USA)) that was used as a control probe to detect polysomy. After deparaffinization of sections, denaturation and hybridization of the probes were carried out following previously described protocols [25]. The experimental results were evaluated through an epifluorescence microscope (**Olympus Corporation of the Americas Headquarters** Corporate Parkway Center Valley, PA, USA); the images were acquired through a CCD microscopy camera.

### 4.4. ALK Gene Rearrangement Interpretation

FISH for *ALK*-R was considered positive, as previously described, when (i) one fusion signal and two separated orange and green signals, as a classic break-apart pattern, or (ii) a single red signal without a corresponding green signal in addition to fused signals, as an atypical pattern, were observed. A case was interpreted as positive when more than 15% of the cells were positive [25].

### 4.5. ALK Copy Number Gain/Amplification Interpretation

The mean copy number for the *ALK* locus (LSI) was evaluated first. The mean copy number of CEP2 was then detected by performing the FISH assay on adjacent serial TMA sections. In order to screen for polyploidy, the ratio between *ALK* gene mean copy number and CEP2 mean copy number was finally evaluated.

The criteria for copy number aberrations of *ALK* gene were the cutoffs proposed by Salido et al.: *ALK*-CNG with a mean copy number of 3 to 5 fusion signals in ≥10% of cells and *ALK*-A with the presence of ≥6 copies of *ALK* per cell in ≥10% of analyzed cells [13]. When clusters were observed, we interpreted cases where more than 10% of cells showed *ALK* clusters as cases of amplification.

Amplification of the *ALK* locus gene was considered to occur when the ALK LSI/CEP2 ratio was ≥2; meanwhile, a case carrying *ALK*-CNG that showed an ALK LSI/CEP2 ratio of <2 after corrections by control probes was interpreted as having chromosomal gains due to polyploidy [17].

### 4.6. Centromeric Alpha-Satellite Specific for Chromosome 2 Interpretation

In a normal interphase nucleus, two orange signals associated to the disomic status of chromosome 2 are expected; an increase in signals per nucleus indicates the polyploidy of the chromosome.

### 4.7. Immunohistochemistry Analysis of ALK Protein Expression

Immunohistochemical staining was performed on 4-μm-thick TMA sections. IHC assays were performed using three different anti-ALK antibodies: ALK1 Ventana, 5A4 Abcam and D5F3 Ventana. IHC using anti-ALK clones ALK1 and 5A4 was performed with ultraView (Ventana Medical Systems Inc., Tucson, AZ, USA), and the scoring was based on the cytoplasmic and/or membrane-staining intensity as follows: strong staining, 3+; moderate staining, 2+; weak staining, 1+ [26].

IHC assays using the VENTANA anti-ALK (D5F3) rabbit monoclonal primary antibody were performed according to the manufacturer’s protocol. The staining results were evaluated according to a binary scoring system (positive or negative) [23].

### 4.8. In Situ Hybridization (ISH) Assay for ALK RNA Detection

In situ detection of ALK RNA was performed using the RNAscope (RNAscope 2.5 HD Detection Reagent—BROWN User Manual, (Advanced Cell Diagnostics USA, Newark, CA) according to the manufacturer’s instructions. The assay was performed using two controls: peptidylprolyl isomerase B (cyclophilin B) (PPIB) mRNA, a positive control and duplex negative control probe (dapB), a negative control. The TMA sections were pretreated using the target retrieval solution (slides were boiled at 95 °C for 30 min) and the protease treatment (slides were treated at 40 °C for 30 min). Then, we hybridized the ALK probe for 2 h at 40 °C. The detection kit (BROWN) was used to amplify and reveal the signal, according to the manufacturer’s instructions. The evaluation of the signals was independently carried out by two different observers.

ALK ISH was interpreted as positive considering brown punctate dot-like signals present in the nucleus and/or cytoplasm. The analysis was carried out on at least 60 tumor cells.

The expression of ALK was analyzed in every core of the TMAs. ALK had an expression level varying between 0 to >6 copies per cell. We sorted the cases into positive (>2 dots/cell in 10% cells) or negative (no staining or 1 dot/cell in 10% cells).

### 4.9. Statistical Analysis

In order to determine the association of clinical and pathological features with ALK protein and/or mRNA expression and *ALK*-CNG or amplification, a Pearson chi-square test was conducted using SPSS 20.0 for Mac (SPSS Inc., Chicago, IL, USA).

## 5. Conclusions

In conclusion, we found that extra copies of the *ALK* gene occurred most frequently because of unspecific chromosome 2. Thus, FISH analysis of chromosome 2 centromeric probes is indispensable in defining true *ALK* copy number gain status and amplification compared to chromosome 2 polysomy.

In neuroblastomas, high ALK expression was found to be associated with *ALK* amplification, as well as copy number gain of the *ALK* locus, supporting the hypothesis that ALK expression depends upon increasing the DNA dose; thus, we could expect a superimposable feature in NSCLC. However, in NSCLC cases harboring *ALK*-A, ALK immunostaining and ALK mRNA ISH are negative. This supports the hypothesis that *ALK*-A is not a productive mutational event in the biological setting of NSCLC because such genetic occurrence is related to the absence of gene transcription rather than to an undetectable protein expression through the common clones used in immunohistochemical routine. Thus, as *ALK*-A has no critical impact on clinical behavior and could not be considered a possible target for specific biological therapy, our results should be confirmed in prospective clinical studies.

## Figures and Tables

**Table 1:** Clinical and pathological features of patients.

Characteristics	No. of Cases (%)
All cases	578
Age	
≥65 years	317 (54.8%)
<65 years	261 (45.2%)
**Gender**	
Male	397 (68.7%)
Female	181 (31.3%)
**Smoking Habit**	
Yes	330 (57.1%)
No	83 (14.4%)
ex-smokers	32 (5.5%)
NA	133 (23.0%)
**Histotype**	
ADCs	398 (68.9%)
SQs	139 (24.0%)
AdSqLCs	5 (0.9%)
Other	36 (6.2%)
**Disease Stage**	
IA	132 (22.8%)
IB	120 (20.8%)
IIA	188 (32.6%)
IIB	23 (4.0%)
III	2 (0.3%)
IV	81 (14.0%)
NA	32 (5.5%)
**Grade**	
I	37 (6.4%)
II	262 (45.3%)
III	210 (36.3%)
IV	2 (0.3%)
NA	67 (11.6%)

NA: not available; ADCs: adenocarcinomas; SQs: squamous cell carcinomas; AdSqLCs: adenosquamous lung carcinomas.

**Table 2:** Clinical and pathological features of patients harboring *ALK* amplification.

Pz *ALK*-A	Gender	Age	Histotype	Stage	Grade	Smoker Status	ALK ISH	ALK IHC	*ALK*-R
1	M	60	SQ	IIA	G3	NA	−	−	WT
2	M	74	ADC	IIA	G2	Y	−	−	WT
3	M	64	SQ	IIA	G2	Y	−	−	WT
4	M	63	ADC	IB	NA	Y	−	−	WT
5	F	66	ADC	IIA	G2	NA	−	−	WT
6	M	77	SQ	IIA	G3	Y	−	−	WT
7	M	55	SQ	IIA	G2	EX	−	−	WT
8	F	71	ADC	IIA	G2	EX	+	+	R
9	M	59	SQ	IIA	G2	EX	−	−	WT
10	M	49	ADC	IIA	G2	NA	−	−	WT
11	M	63	ADC	IA	G3	NA	−	−	WT
12	F	64	ADC	IV	G3	NA	−	−	WT
13	M	63	ADC	IB	G2	Y	−	−	WT
14	M	67	ADC	IIA	G2	Y	−	−	WT
15	F	70	SQ	IV	G3	EX	−	−	WT
16	M	63	ADC	IA	G3	Y	−	−	WT
17	M	70	ADC	IIA	G2	NA	−	−	WT

Anaplastic lymphoma kinase (ALK); NA: not available; ADCs: adenocarcinomas; SQs: squamous cell carcinomas.

**Table 3:** Cases harboring *ALK* aberrations in FISH.

*ALK* FISH Status	ALK ISH+	ALK IHC+
18 *ALK*-R	18/18	18/18
17 *ALK*-A	1/17	1/17
14 *ALK*-CNG	0/14	0/14
72 *ALK*-CNG and Polisomy Chr2	0/72	0/72

*ALK*-R: *ALK* rearrangement; *ALK*-A: *ALK* amplification; *ALK*-CNG: *ALK* copy number gain, 3 to 5 fusion signals; FISH: fluorescence in situ hybridization; ISH: in situ hybridization; IHC: immunohistochemistry.

## Abbreviations

ALK	anaplastic lymphoma kinase
NSCLC	non-small cell lung cancer
*ALK*-CNG	*ALK* gene copy number gain
*ALK*-R	*ALK* gene rearrangement
*ALK*-A	*ALK* gene amplification
FISH	fluorescence in situ hybridization
ALCL	anaplastic large-cell lymphoma
JAK/STAT	Janus-activated kinase/signal transducer and activator of transcription
EGFR	epidermal growth factor receptor
ADC	adenocarcinoma
SQ	squamous carcinoma
AdSqLC	adenosquamous lung carcinomas
TMA	tissue microarrays
CEP2	centromeric probe 2
